# Supplementary material for: Visualizing the membrane disruption action of antimicrobial peptides by cryo-electron tomography
Source: Nat Commun. 2023 Sep 6;14:5464. doi: 10.1038/s41467-023-41156-2 (PMC10482868; doi:10.1038/s41467-023-41156-2)

Visualizing the membrane disruption action of antimicrobial peptides by cryo-electron tomography

Eric H.-L. Chen[1#], Chun-Hsiung Wang[1#], Yi-Ting Liao[1#], Feng-Yueh Chan[2], Yui Kanaoka[2], Takayuki Uchihashi[2, 3, 4], Koichi Kato[4, 5, 6], Longsheng Lai[7], Yi-Wei Chang[7], Meng-Chiao Ho[1, 8*], Rita P.-Y. Chen[1, 8*]

Supplementary Materials

Supplementary Fig. 1 Fluorescent leakage assay

Supplementary Fig. 2 CryoET analysis of an intact minicells.

Supplementary Fig. 3 The cryo-tomographic slices and one 3-D segmentation of the minicells treated with 2% Triton X-100.

Supplementary Fig. 4 Comparison of pepD2M and melittin. (a) pore formation; (b) shrunken cell formation; (c) lipid cluster.

Supplementary Movie 1_ Reconstructed cryo-ET 3D tomograms of an intact minicell (Supplementary Fig. 2)

Supplementary Movie 2_ Reconstructed cryo-ET 3D tomograms of a minicell treated with pepD2M (Fig. 3a to e)

Supplementary Movie 3_ Reconstructed cryo-ET 3D tomograms of a minicell treated with pepD2M (Fig. 2h)

Supplementary Movie 4_ Time-dependent AFM images of *E.coli* lipids treated with pepD2M (Fig. 4a)

Supplementary Movie 5_ Time-dependent AFM images of *E.coli* lipids treated with

pepD2M (Fig. 4b), starting from 460s.

Supplementary Movie 6_ Time-dependent AFM images of *E.coli* lipids treated with pepD2M (Fig. 4d)

Supplementary Movie 7_ Reconstructed cryo-ET 3D tomograms of a minicell treated with melittin (Fig. 5b)

Supplementary Movie 8_ Reconstructed cryo-ET 3D tomograms of a minicell treated with melittin (Fig. 5c)

Supplementary Movie 9_ Time-dependent AFM images of *E.coli* lipids treated with melittin (Fig. 6a)

Supplementary Movie 10_ Reconstructed cryo-ET 3D tomograms of a minicell treated with Triton X-100 (Supplementary Fig. 3a)

Experimental methods

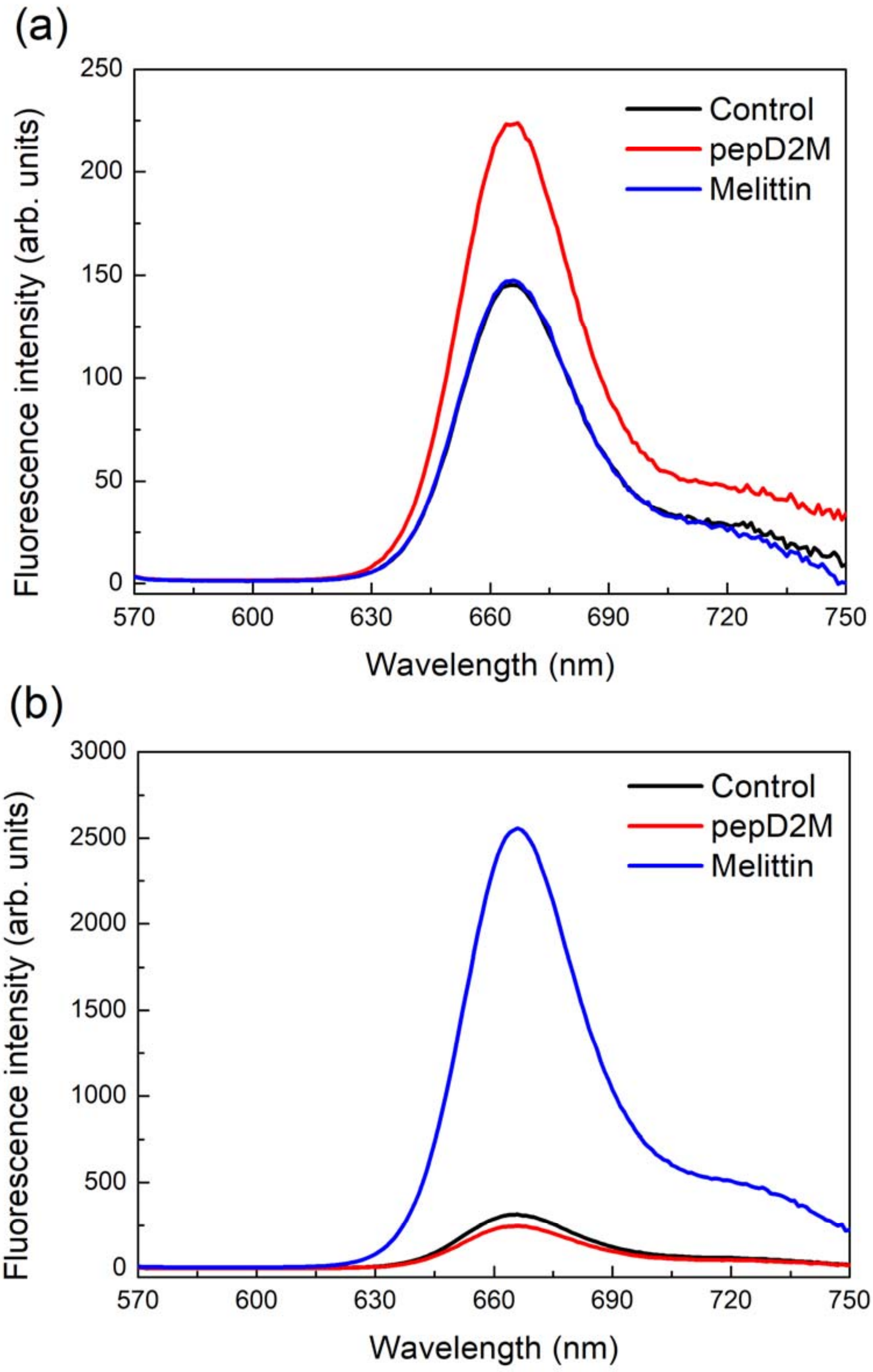

Supplementary Fig. 1 Fluorescent leakage assay. (a) The fluorescence spectra of the PE/PG liposomes treated by water (control), pepD2M, and melittin. (b) The fluorescence spectra of the DOPC liposomes treated by water (control), pepD2M, and melittin.

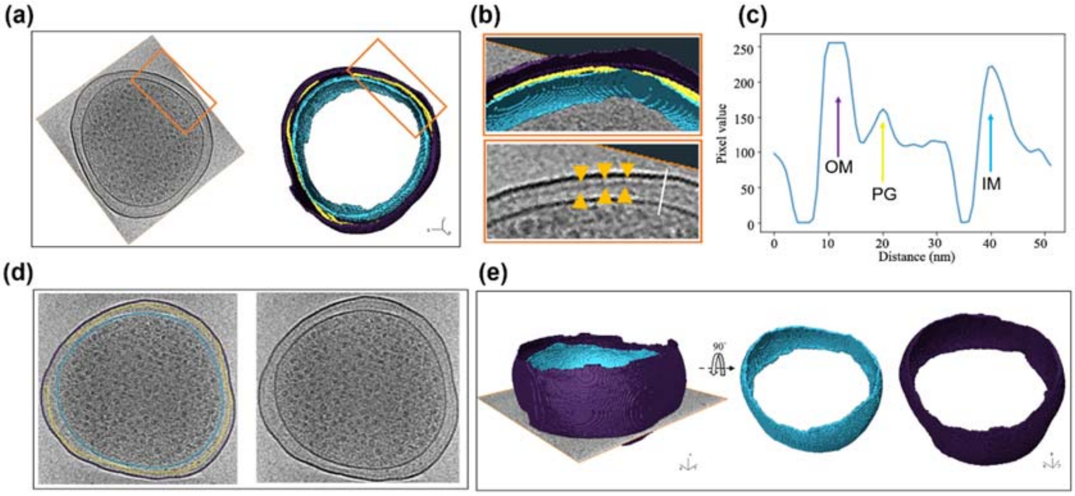

Supplementary Fig. 2 CryoET analysis of an intact minicell. (a) Cryo-tomographic slices (left) and the corresponding segmentations (right). (b) Magnified view of the orange box in (a). The arrows indicate the PG (yellow) located between the OM (violet) and IM (cyan). (c) The line profile plot of the inverted pixel values indicated by a white line in (b) shows the OM, PG, and IM spacing. (d) A tomographic slice (right). The OM, PG, and IM layers are colored (left). (e) 3D tomogram segmentations of an intact minicell.

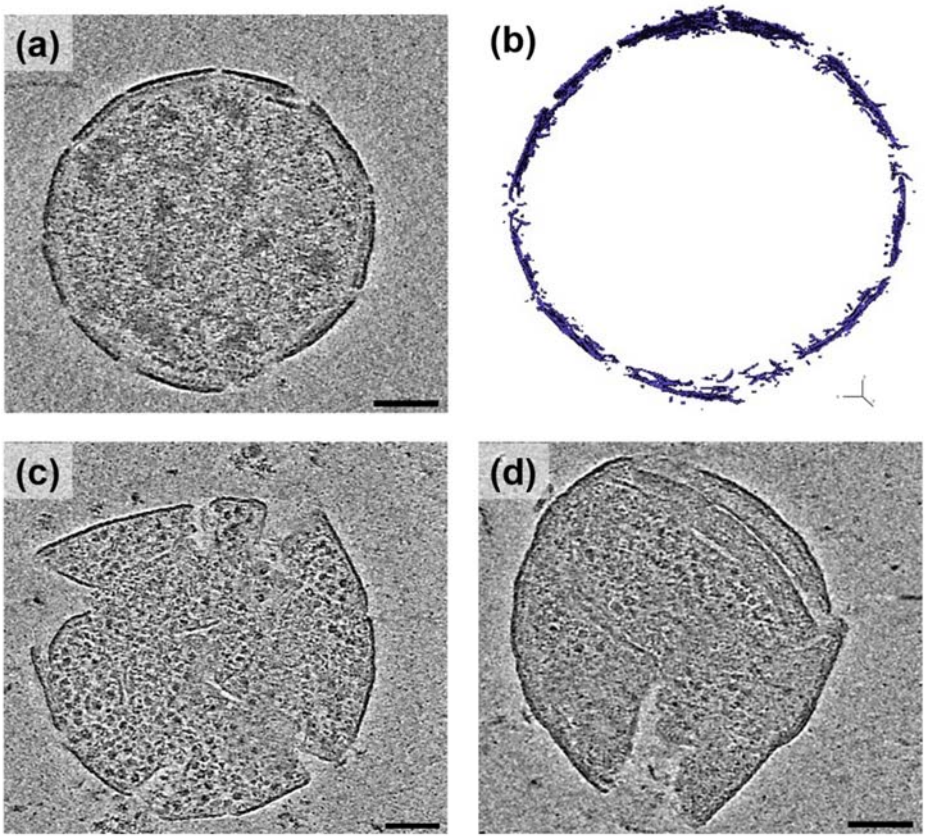

Supplementary Fig. 3 The cryo-tomographic slices and one 3-D segmentation of the minicells treated with 2% Triton X-100. (a, c, d) The cryo-tomographic slices. (b) The 3-D segmentation of (a). OM: violet. Scale bar = 100 nm.

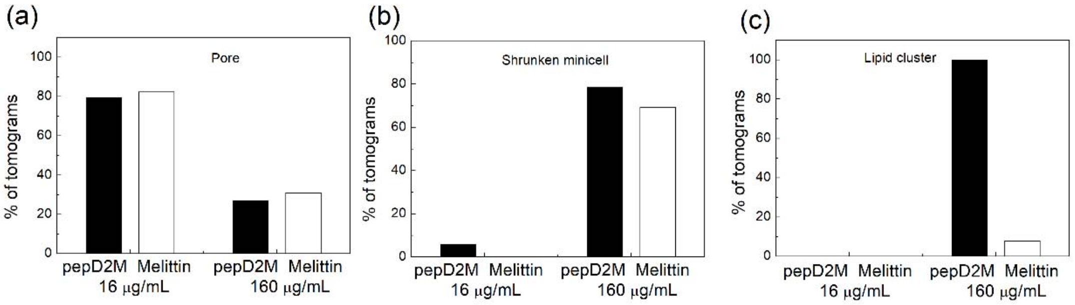

Supplementary Fig. 4 Comparison of pepD2M and melittin. (a) pore formation; (b) shrunken cell formation; (c) lipid cluster.

Experimental methods

Liposome preparation

DOPC (1,2-dioleoyl-sn-glycero-3-phosphocholine), POPE (1-palmitoyl-2-oleoyl-sn-glycero-3-phosphoethanolamine) and DOPG (1,2-dioleoyl-sn-glycero-3-[phospho-rac-(1-glycerol) were purchased from Avanti Polar Lipids (USA). DOPC and the mixture of POPE/DOPG (1:1, w/w) were individually dissolved in chloroform/methanol (9/1, v/v) in a glass tube. The solvents were evaporated through nitrogen purging, forming a thin lipid film on the glass surface. Four hundred microliters of deionized water or buffer (20 mM phosphate buffer, 100 mM NaCl, pH 7) were added and mixed. Five freeze-thaw cycles were conducted, and then the mixture was extruded through a polycarbonate filter (with a 200-nm pore size) using an Avanti Mini-Extruder (Avanti Polar Lipids, USA) to obtain liposomes.

Circular dichroism spectroscopy

PepD2M was dissolved in water to make a stock solution (1.28 mg/mL). This peptide was added to water or the liposome solutions to a final peptide concentration of 60 μg/mL. The CD spectra between 190 and 260 nm were recorded on a J-815 CD spectrometer (JASCO, Japan). Each sample was scanned twice with a bandwidth of 1 nm and a step resolution of 0.1 nm.

Dye leakage experiment

PepD2M and melittin were dissolved in water to make a stock solution. Cyanine-5 (Cy5) was purchased from General Electric (USA) and dissolved in DI to make a stock solution (1mg/mL). DOPC and PE/PG liposomes were prepared in water containing Cy5 (1 mg/mL). The free dye not trapped in the liposomes was removed using the PD-10 column (General Electric, USA). The Cy5-trapped liposomes were

concentrated using Amicon Ultra (10 kDa cut-off, Merck, Taiwan) at 1,000 g for 90 min. PepD2M and melittin were added to the liposomes to the final 20 μg/mL peptide concentration. After 5 min, the peptide-treated liposomes were spun using Amicon Ultra (10 kDa cut-off, Merck, Taiwan) at 10,000 g for 30 min. The released dye was in the filtrate. The fluorescence spectrum of the filtrate was recorded from 570 nm to 750 nm on a spectrofluorometer (FP-8300 Jasco, Japan).

Pixels used in the image recording of HS-AFM

16 μg/mL of pepD2M (movie 4): 500 × 500 nm^2 at 256 × 256 pixels.

16 μg/mL of pepD2M (movie 5): 400 × 400 nm^2 at 200 × 200 pixels.

16 μg/mL of pepD2M (movie 6): 800 × 800 nm^2 at 400 × 400 pixels.

4 and 8 μg/mL of pepD2M: 400 × 400 nm^2 at 200 × 200 pixels.

4 , 8 and 16 μg/mL (Movie 9) of melittin: 400 × 400 nm^2 at 200 × 200 pixels.

HS-AFM image processing

The images were processed and analyzed with a custom-made tool based on Igor Pro 9 (https://www.wavemetrics.com/products/igorpro). The acquired AFM raw images were loaded into the software, then subtracting the tilted background using a linear plane function. The image drift in X and Y directions during imaging was corrected by image correlation techniques with static features identified in the image, such as consistent lipid patch boundary or immobilized micelles on the mica or lipid surface. Meanwhile, we did not compensate for drift in the Z direction during the AFM imaging. However, since the dimple depth is measured with respect to the lipid membrane surface, even if there is a drift in the Z direction, we consider that it does not significantly affect the relative height change because the imaging speed is fast enough

compared to the drift to tilt the entire image.

HS-AFM image analysis

As the first step of image analysis, the pixels corresponding to the pores were defined. On the flattened images, the lipid height was determined relative to the mica surface. We then manually defined a height threshold based on the visual appearance of the pores, such that only pixels with heights lower than this threshold were considered to correspond to the pores. The area of the pores was calculated by summing up the number of pixels with a height lower than the threshold. Then, the lowest value determined from the site of the pores was defined as the depth of the pore.

Cryo-ET processing workflow

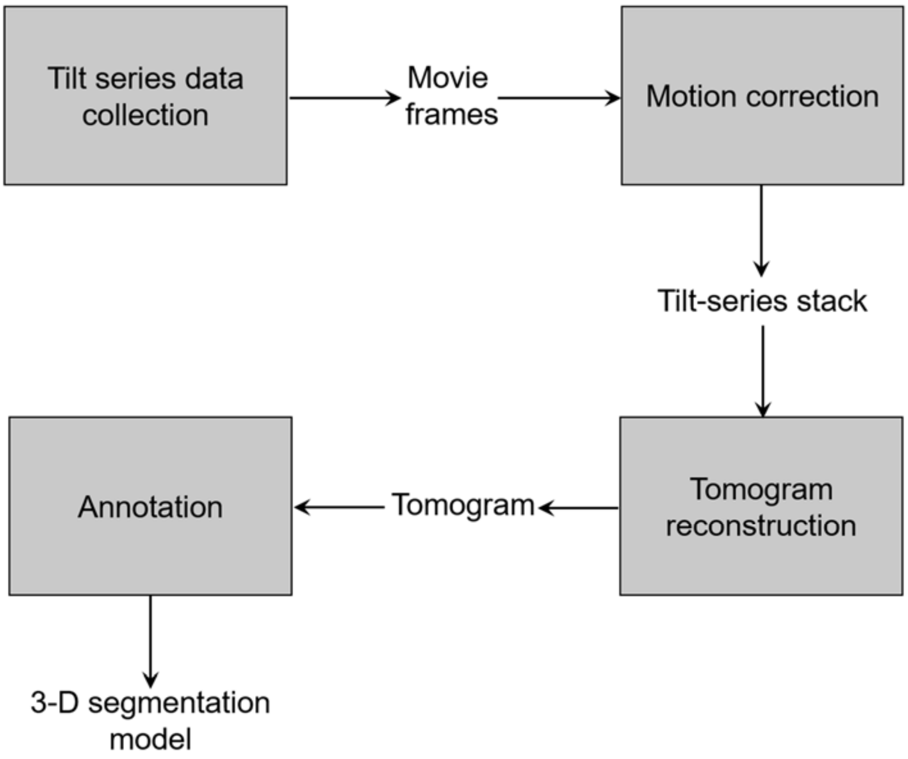

Supplement: Supplementary file 1 — Supplementary Information [file 41467_2023_41156_MOESM1_ESM.pdf]
