# Supplementary material for: Visualizing the membrane disruption action of antimicrobial peptides by cryo-electron tomography
Source: Nat Commun. 2023 Sep 6;14:5464. doi: 10.1038/s41467-023-41156-2 (PMC10482868; doi:10.1038/s41467-023-41156-2)
Supplement: Supplementary file 3 — Description of Additional Supplementary Files [file 41467_2023_41156_MOESM3_ESM.pdf]

Description of Additional Supplementary Files

File Name: Supplementary Movie 1
Description: Reconstructed cryo-ET 3D tomograms of an intact minicell (Supplementary Fig. 2)

File Name: Supplementary Movie 2
Description: Reconstructed cryo-ET 3D tomograms of a minicell treated with pepD2M (Fig. 3a to e)

File Name: Supplementary Movie 3
Description: Reconstructed cryo-ET 3D tomograms of a minicell treated with pepD2M (Fig. 2h)

File Name: Supplementary Movie 4
Description: Time-dependent AFM images of E.coli lipids treated with pepD2M (Fig. 4a)

File Name: Supplementary Movie 5
Description: Time-dependent AFM images of E.coli lipids treated with 2 pepD2M (Fig. 4b), starting from 460s.

File Name: Supplementary Movie 6
Description: Time-dependent AFM images of E.coli lipids treated with pepD2M (Fig. 4d)

File Name: Supplementary Movie 7
Description: Reconstructed cryo-ET 3D tomograms of a minicell treated with melittin (Fig. 5b)

File Name: Supplementary Movie 8
Description: Reconstructed cryo-ET 3D tomograms of a minicell treated with melittin (Fig. 5c)

File Name: Supplementary Movie 9
Description: Time-dependent AFM images of E.coli lipids treated with melittin (Fig. 6a)

File Name: Supplementary Movie 10
Description: Reconstructed cryo-ET 3D tomograms of a minicell treated with Triton X-100 (Supplementary Fig. 3a)
