# Supplementary material for: Visualizing the membrane disruption action of antimicrobial peptides by cryo-electron tomography
Source: Nat Commun. 2023 Sep 6;14:5464. doi: 10.1038/s41467-023-41156-2 (PMC10482868; doi:10.1038/s41467-023-41156-2)
Supplement: Supplementary file 14 — Reporting Summary [file 41467_2023_41156_MOESM14_ESM.pdf]

nature portfolio

Corresponding author(s): NCOMMS-22-26313B

Last updated by author(s): Jul 26, 2023

Reporting Summary

Nature Portfolio wishes to improve the reproducibility of the work that we publish. This form provides structure for consistency and transparency in reporting. For further information on Nature Portfolio policies, see our Editorial Policies and the Editorial Policy Checklist.

Statistics

For all statistical analyses, confirm that the following items are present in the figure legend, table legend, main text, or Methods section.

n/a	Confirmed	
☐	☒	The exact sample size (*n*) for each experimental group/condition, given as a discrete number and unit of measurement
☐	☒	A statement on whether measurements were taken from distinct samples or whether the same sample was measured repeatedly
☐	☒	The statistical test(s) used AND whether they are one- or two-sided<br>*Only common tests should be described solely by name; describe more complex techniques in the Methods section.*
☒	☐	A description of all covariates tested
☒	☐	A description of any assumptions or corrections, such as tests of normality and adjustment for multiple comparisons
☒	☐	A full description of the statistical parameters including central tendency (e.g. means) or other basic estimates (e.g. regression coefficient) AND variation (e.g. standard deviation) or associated estimates of uncertainty (e.g. confidence intervals)
☐	☒	For null hypothesis testing, the test statistic (e.g. *F*, *t*, *r*) with confidence intervals, effect sizes, degrees of freedom and *P* value noted<br>*Give P values as exact values whenever suitable.*
☒	☐	For Bayesian analysis, information on the choice of priors and Markov chain Monte Carlo settings
☒	☐	For hierarchical and complex designs, identification of the appropriate level for tests and full reporting of outcomes
☒	☐	Estimates of effect sizes (e.g. Cohen's *d*, Pearson's *r*), indicating how they were calculated

Our web collection on statistics for biologists contains articles on many of the points above.

Software and code

Policy information about availability of computer code

Data collection	Tomography-4.10.0 for Talos Arctica and Tomography-5.2.0 for Titan Krios.
Data analysis	3D tomogram reconstruction: Inspect3D-4.2 software package. Tomogram denoise: Topaz v0.2.5. Tomogram segmentation: Amira 2022.1. Movie generation: IMOD-4.9.12 and Amira 2022.1. The pixel value measurement: FIJI/ImageJ 1.52r. The HS-AFM images were analyzed with a custom-made tool based on Igor Pro 9. Statistic analysis of Figures 2f and 7 and Supplementary Fig. 2c : Numby/Matplotlib/Seaborn libraries in Python 3.5. Bar plot in Figure 1, 2m, 2n, 5g, 5h, and Supplementary Fig. 1 and 4 : Origin 7.5.

For manuscripts utilizing custom algorithms or software that are central to the research but not yet described in published literature, software must be made available to editors and reviewers. We strongly encourage code deposition in a community repository (e.g. GitHub). See the Nature Portfolio guidelines for submitting code & software for further information.

Data

Policy information about availability of data

All manuscripts must include a data availability statement. This statement should provide the following information, where applicable:

- Accession codes, unique identifiers, or web links for publicly available datasets
- A description of any restrictions on data availability
- For clinical datasets or third party data, please ensure that the statement adheres to our policy

The data generated in this study are provided in the article and the supplementary information. The raw data of Figs. 1, 2, 5, and 7 and Supplementary Figs. 1, 2,

March 2021

and 4 are provided in the Source Data file. Source data are provided in this paper. The cryo-ET images are deposited in Figshare (https://figshare.com/s/311c7b0c9461ed67cd36).

Human research participants

Policy information about studies involving human research participants and Sex and Gender in Research.

Reporting on sex and gender	NA
Population characteristics	N/A
Recruitment	N/A
Ethics oversight	N/A

Note that full information on the approval of the study protocol must also be provided in the manuscript.

Field-specific reporting

Please select the one below that is the best fit for your research. If you are not sure, read the appropriate sections before making your selection.

☒ Life sciences ☐ Behavioural & social sciences ☐ Ecological, evolutionary & environmental sciences

For a reference copy of the document with all sections, see nature.com/documents/nr-reporting-summary-flat.pdf

Life sciences study design

All studies must disclose on these points even when the disclosure is negative.

Sample size	For cryo-ET of pepD2M, 34, 37, and 52 tomograms were analyzed for 16, 160, and 640 μg/mL pepD2M, respectively. For cryo-ET of melittin, 51, 37, and 52 tomograms were analyzed for 16, 64, and 160 μg/mL melittin, respectively.<br>For HS-AFM, four to five observations were made from freshly prepared samples for each peptide concentration. Recorded pore number is marked for each peptide concentration in Fig. 7bc.
Data exclusions	No data exclusion
Replication	For cryo-ET, images were recorded from freshly prepared samples two to three times for each peptide concentration. For HS-AFM, four to five observations were made from freshly prepared samples for each peptide concentration.
Randomization	N/A
Blinding	N/A

Reporting for specific materials, systems and methods

We require information from authors about some types of materials, experimental systems and methods used in many studies. Here, indicate whether each material, system or method listed is relevant to your study. If you are not sure if a list item applies to your research, read the appropriate section before selecting a response.

Materials & experimental systems

n/a	Involved in the study	
☒	☐	Antibodies
☒	☐	Eukaryotic cell lines
☒	☐	Palaeontology and archaeology
☒	☐	Animals and other organisms
☒	☐	Clinical data
☒	☐	Dual use research of concern

Methods

n/a	Involved in the study	
☒	☐	ChIP-seq
☒	☐	Flow cytometry
☒	☐	MRI-based neuroimaging
